# Selection of the sex‐linked *inhibitor of apoptosis* in mountain pine beetle (*Dendroctonus ponderosae*) driven by enhanced expression during early overwintering

**DOI:** 10.1002/ece3.4164

**Published:** 2018-05-24

**Authors:** Linda C. Horianopoulos, Celia K. Boone, G. D. N. Gayathri Samarasekera, Gurkirat K. Kandola, Brent W. Murray

**Affiliations:** ^1^ Natural Resources and Environmental Studies Institute University of Northern British Columbia Prince George BC Canada

**Keywords:** *inhibitor of apoptosis*, microsatellites, neo‐X/neo‐Y evolution, selection

## Abstract

The mountain pine beetle (*Dendroctonus ponderosae*) is an insect native to western North America; however, its geographical range has recently expanded north in BC and east into Alberta. To understand the population structure in the areas of expansion, 16 gene‐linked microsatellites were screened and compared to neutral microsatellites using outlier analyses of *F*_st_ and *F*_ct_ values. One sex‐linked gene, *inhibitor of apoptosis* (*IAP*), showed a strong signature of positive selection for neo‐X alleles and was analyzed for evidence of adaptive variation. Alleles of *IAP* were sequenced, and differences between the neo‐X and neo‐Y alleles were consistent with neutral evolution suggesting that the neo‐Y allele may not be under functional constraints. Neo‐Y alleles were amplified from gDNA, but not effectively from cDNA, suggesting that there was little *IAP* expression from neo‐Y alleles. There were no differences in overall *IAP* expression between males and females with the common northern neo‐X allele suggesting that the neo‐X allele in males compensates for the reduced expression of neo‐Y alleles. However, males lacking the most common northern neo‐X allele thought to be selected for in northern populations had reduced overall *IAP* expression in early October—at a time when beetles are preparing for overwintering. This suggests that the most common allele may have more rapid upregulation. The reduced function of neo‐Y alleles of *IAP* suggested by both sequence differences and lower levels of expression may foster a highly selective environment for neo‐X alleles such as the common northern allele with more efficient upregulation.

## 1 INTRODUCTION

**Figure 1:**
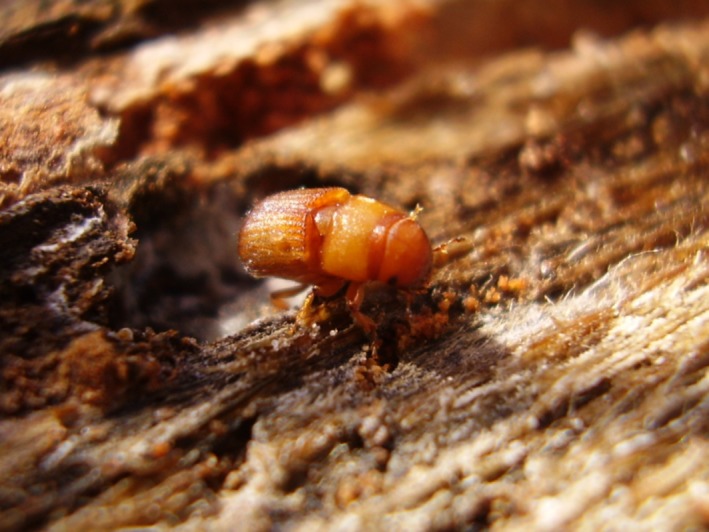
A teneral adult mountain pine beetle (*Dendroctonus ponderosae*)

The mountain pine beetle (*Dendroctonus ponderosae*, Figure 1) is native to North American forests, but during epidemics this insect can devastate pine forests at the landscape level (Safranyik & Carroll, 2006). The frequency, duration, and severity of outbreaks are influenced by the availability of susceptible host trees and by the suitability of the climate (Taylor, Carroll, Alfaro, & Safranyik, 2006). Climate has traditionally been considered the main constraint on the range of the beetles in North America because under bark temperatures of less than −40°C are fatal to the mountain pine beetle (Carroll, Taylor, Regniere, & Safranyik, 2003). As epidemics can have severe ecological and economic impacts, it is important to model and understand mountain pine beetle outbreaks to inform future forest management practices.

The devastation caused during an epidemic is no longer limited to the historic range of *D. ponderosae*, but is now also a concern in new habitats where the beetles have spread in recent outbreaks. This includes movement into higher elevations in the United States, north and east in Canada, and even into a novel host, that is, jack pine (*Pinus banksiana*), in northern Alberta (Cullingham et al., 2011). As the mountain pine beetle has moved into these new areas, surveying the genetic diversity of beetles throughout their range has been useful in identifying both the spatial genetic structure (Samarasekera et al., 2012) and the genes potentially under selection in the expanding range (Janes et al., 2014). The identification of genes that are under selection at the northeastern limits of the beetles range may provide insights into how they have adapted to overcome the defenses of a novel host as well as the cold climates of these environments.

In looking for adaptive variation, microsatellites or Simple Tandem Repeats (STRs) are often overlooked in favor of other types of variation such as Single Nucleotide Polymorphisms (SNP's). However, microsatellites can be a useful tool to identify genes under selection that may provide local adaptation. This is especially true of Expressed Sequence Tags (ESTs) which have variable repeat lengths within the transcript which may directly result in functional differences and are easy to track through genotyping. Comparing variation in gene‐linked microsatellites to the variation in neutral microsatellites within the same populations can effectively identify outliers. These outliers may be under selection in populations where certain alleles of the associated gene provide a local adaptation causing allele frequencies to be different than would be expected based on the distribution of neutral markers (Holderegger et al., 2008; Meier, Hansen, Bekkevold, Skaala, & Mensberg, 2011).

In addition to being useful markers to identify genes under selection, microsatellites may provide local adaptation by creating variation in the expression or function in different alleles of the gene (Li, Korol, Fahima, & Nevo, 2004). The expression of a gene can be greatly affected by the number of repeat units in a microsatellite (Gemayel, Cho, Boeynaems, & Verstrepen, 2012; Gymrek et al., 2016). This is particularly true if the microsatellite is in a promoter region or transcription factor binding site. Furthermore, if the microsatellite is in between two transcription factor binding sites, variation in the repeat number may interrupt or promote interactions between transcription factors (Li et al., 2004). Finally, microsatellites have been shown to inhibit nucleosome formation resulting in an open chromatin structure around microsatellites thereby promoting transcription (Gemayel et al., 2012). Variation in repeat number of a microsatellite in the coding sequence may also affect the proper formation or function of the resulting protein. In particular, microsatellites can introduce frameshift mutations, amino acid repeats can result in protein aggregation, and variable numbers of amino acids can affect protein binding interactions (Gemayel et al., 2012).

In a previous study, Samarasekera, Keeling, Bohlmann, and Murray (2011) identified 50 polymorphic gene‐linked microsatellites as a resource to investigate local adaptations in mountain pine beetle populations. These were used to identify outliers showing signatures of selection. If these genes do provide a selective advantage, the location and repeat motif of the microsatellite may be able to inform what type of advantage is being conferred. As the population is expanding northeastwardly into ranges with colder climates, we hypothesize that alleles under positive selection may facilitate cold tolerance. Our screen identified the sex‐linked gene for *inhibitor of apoptosis* (*IAP*) which has a microsatellite in the coding sequence as a gene showing signatures of positive selection. This gene was previously found to be upregulated overall during overwintering by mountain pine beetle larvae (Robert et al., 2016). Subsequent analysis of variation in expression levels among beetles with different genotypes during early overwintering in a northern population suggests that there are elevated levels of transcription in early overwintering in larvae with certain genotypes which may be driving this positive selection. Furthermore, the male‐specific alleles of this sex‐linked gene were not detected in cDNA and the sequences were divergent from neo‐X linked alleles in a manner consistent with neutral evolution. Mountain pine beetle possesses a relatively recently evolved neo‐X/Y sex chromosome shared with only one other species, the Jeffrey pine beetle, in the genus *Dendroctonus* (Zúñiga, Cisneros, Hayes, & Macias‐Samano, 2002). Our findings suggest that the male‐specific allele was no longer functional. Therefore, having one functional allele may have resulted in a stronger selective pressure on the neo‐X alleles in male beetles. This is one example of local adaptation that may be facilitating the expansion of the range of mountain pine beetle to colder climates.

## 2 MATERIALS AND METHODS

### 2.1 Developing and screening a gene‐linked microsatellite database in western Canada

Individual beetles collected from six sampling locations representing at least two subpopulations of MPB in Western Canada (North: Houston, BC; Mackenzie, BC; Grande Prairie, AB, and South: AB; Green Lake, BC; Banff, AB; Nancy Greene, BC [Samarasekera et al., 2012]) were genotyped at 16 gene‐linked microsatellites (Table 1). Each of these microsatellites is associated with a unique scaffold (Supporting Information Table S1), and there is no evidence of linkage disequilibrium in either the neutral microsatellites (Samarasekera et al., 2012) or the gene‐linked microsatellites used (Samarasekera et al., 2011). M13 tailed primers were used to amplify polymorphic microsatellites with VIC, NED, PET, or FAM fluorescent labels on the amplicons (Life Technologies Inc., Burlington, ON, Canada). The primer sequences were those developed by Samarasekera et al. (2011) with the appropriate 5′ tails. The loci were amplified using either the touchdown PCR protocol described by Samarasekera et al. (2011) or by multiplex PCR using Qiagen Multiplex PCR kit (Qiagen Inc., Toronto, ON, Canada; Table 1). The thermocycling conditions used for multiplexed loci were: 95°C for 15 min, 27 cycles of 94°C for 30 s, 58°C for 90 s, and 72°C for 60 s, and a final extension step at 60°C for 30 min. Fragment analysis was performed on PCR products using ABI 3130xL and GeneScan 500 LIZ size standard following the manufacturer's protocol (Life Technologies). Amplicon size was scored using GeneMapper v4.0 (Life Technologies). Genotypes were added to a database of 14 neutral microsatellite markers (Samarasekera et al., 2012) and used to determine allele frequencies, *F*_st_ values, *F*_ct_ values, and to identify outliers as candidates for selection using GenAlEx v6.5 (Peakall & Smouse, 2006, 2012), Arlequin v3.5 (Excoffier & Lischer, 2010), and Bayescan v2.1 (Foll & Gaggiotti, 2008). A neutral microsatellite previously identified as being sex‐linked, Dpo486, was used for comparison to identify sex‐linked microsatellites (Davis et al., 2009).

**Table 1:** A description of each gene‐linked microsatellite including the repeat motif, location within the transcript, and the PCR method used for each of the gene‐linked microsatellites screened for signatures of selection. The gene with the closest alignment from BLASTx and the organism the aligned gene is from are listed

Microsatellite	Repeat motif	Location	PCR method	Description (BLASTx) – Predicted similar to
MPBC8_7725	TAA	5′	Multiplex	MOB kinase activator‐like 4 (*Pseudomyrmex gracilis*)
MPBC5_6124	AGG	CDS	Touchdown	WD repeat‐containing protein 55 homolog (*Tribolium castaneum*)
MPBC5_811	CTC	CDS	Multiplex	Uncharacterized protein LOC111506581 (*Leptinotarsa decemlineata*)
MPBC6_675	TAG	CDS	Multiplex	Death‐associated inhibitor of apoptosis 2 isoform X2 (*Anoplophora glabripennis*)
MPBC6_7245	TGC	CDS	Multiplex	Prefoldin subunit 5 (*Leptinotarsa decemlineata*)
MPBC7_548	GTG	CDS	Multiplex	Obscurin isoform X9 (*Leptinotarsa decemlineata*)
MPBC8_2778	CAG	CDS	Touchdown	No significant hit
MPBC8_4511	TCA	CDS	Touchdown	PREDICTED: zinc fin ger protein OZF‐like isoform X2 (*Branchiostoma belcheri*)
MPBC8_6649	TCA	CDS	Multiplex	No significant hit
MPBC8_9094	CAT	CDS	Multiplex	PREDICTED: WAS protein family homolog (*Nicrophorus vespilloides*)
MPBC8_9385	CTA	CDS	Multiplex	No significant hit
MPBC5_6823	AAT	CDS	Touchdown	Ornithine decarboxylase antizyme 1‐like protein (*Tribolium castaneum*)
MPBC8_884	GTA	3′	Touchdown	F‐actin‐capping protein subunit alpha (*Anoplophora glabripennis*)
MPBC8_12800	AAC	3′	Multiplex	Antennae‐rich cytochrome P450 (*Tribolium castaneum*)
MPBC5_4357	(TA)_2_TTC(TA)_7_	3′	Touchdown	Ring finger protein 141 (*Anoplophora glabripennis*)
MPBC7_24	(TA)_3_TG(TA)_8_	3′	Touchdown	Chitin deacetylase 1 precursor (*Tribolium castaneum*)

### 2.2 Analysis of North American database

A database of 14 neutral microsatellites and one gene‐linked microsatellite, MPBC6_675 (*inhibitor of apoptosis*,* IAP*)*,* was developed using beetles from 52 populations across western North America (Boone and Murray, unpublished data). This database was then divided into a female‐only and male‐only database because there were distinct neo‐X and neo‐Y linked microsatellites. The female‐only database was examined for *F*_st_ and *F*_ct_ outliers and to determine the distribution of neo‐X allele frequencies in North America. The male database was used to determine the neo‐Y allele frequencies in the same 52 populations. Analysis of the genotypic data was conducted using GenAlEx v6.5 (Peakall & Smouse, 2006, 2012), Arlequin v3.5 (Excoffier & Lischer, 2010), and BayeScan v2.1 (Foll & Gaggiotti, 2008).

### 2.3 Sequencing of *IAP* alleles

DNA was extracted from individual beetles using the Qiagen AllPrep DNA/RNA Mini Kit following the manufacturer's protocols (Qiagen), and *IAP* was amplified in a 10‐μl reaction using the Qiagen Multiplex PCR kit (Qiagen): 1X Qiagen Multiplex PCR Master Mix; 200 nM of forward primer (94F: 5′‐AGCGAGACAGACAACAGCAA‐3′); 200 nM of one of two reverse primers (1209R: 5′‐GGGGAGAAAAGTTCATGTTGA‐3′ or 1320R: 5′‐GCTCGGACCGTGGCTTCT‐3′); and 1 μl of template DNA. Thermocycling conditions were 95°C for 15 min, 30 cycles of 94°C for 30 s, 52°C for 90 s, and 72°C for 90 s, and a final extension step at 72°C for 10 min. Amplicons were run on a 0.8% agarose gel and extracted using GeneJET Gel extraction kit following the manufacturer's protocol (Life Technologies).

Amplified DNA was quantified using the Qubit fluorometric assay for dsDNA (Life Technologies), cloned into a pGEM‐T Easy vector system (Promega, Madison, WI, USA), and transformed into DH5α *E. coli* (Life Technologies). The plasmids were extracted from these using a QiaPrep Miniprep Kit following the manufacturers protocol (Qiagen). Plasmids were screened for the neo‐X and neo‐Y alleles of *IAP* using the primers for the MPBC6_675 microsatellite and the same PCR conditions described earlier for multiplex PCR. Selected plasmid inserts were sequenced using an ABI 3130xL with T7 and SP6 primers (Life Technologies). Sequences were aligned using CodonCode Aligner (v4.2.4), and the dN/dS ratios between the sequences of the neo‐X and neo‐Y alleles were determined using MEGA6 (Tamura, Stecher, Peterson, Filipski, & Kumar, 2013).

### 2.4 Sample collection for *IAP* expression study

Larvae collected from Tête Juane Cache, BC in the autumn of 2008 as part of another study (Fraser, Bonnett, Keeling, & Huber, 2017) were used for expression studies of *IAP*. From this collection, we used larvae collected on October 3 and October 10 as these represent the time‐points immediately before and after the temperature dropped below 0°C. Briefly, upon removal from the tree, larvae were immediately frozen in 1.5 ml tubes using liquid nitrogen, transported to UNBC on dry ice, and stored at −80°C until use. Samples used solely for comparison between neo‐X and neo‐Y expression were collected from four populations: Utah, California, Cypress Hills, and Arizona, and sent to UNBC live. Utah samples were larvae collected in June 2012, California samples were larvae collected in July 2012, Cypress Hills samples were larvae collected in November 2010, and Arizona samples were larvae, pupae, and adults collected in June 2012.

### 2.5 Total RNA and DNA extraction and cDNA synthesis

The RNA and DNA from individual larvae were extracted using a Qiagen AllPrep DNA/RNA Mini Kit following the manufacturer's animal tissue protocol with optional DNase treatment (Qiagen). The amount and purity of RNA was evaluated using a Nanodrop ND‐100 (Nanodrop Technologies Inc., Wilmington, DE, USA). The integrity of RNA was visually inspected using Experion™ RNA StdSens Analysis Kit following the manufacturer's protocol (Bio‐Rad, Hercules, CA, USA). All RNA and DNA samples were stored at −80°C after extraction until required for downstream applications. The RNA extracted from each individual larva was converted to cDNA using a High Capacity cDNA Reverse Transcription Kit following the manufacturer's without RNase inhibitor protocol (Life Technologies). The total amount of RNA used per cDNA synthesis reaction was normalized to be 100 ng of RNA per reaction based on the concentration estimate from the Nanodrop ND‐100. The cDNA was stored at −80°C until required for downstream applications.

### 2.6 Genotyping of *IAP* and *prefoldin subunit 5*

The microsatellite regions of the *IAP* and another sex‐specific microsatellite region in *prefoldin subunit 5* were amplified to determine the genotype of each larva and confirm its sex. These two loci were amplified in a duplex PCR using Qiagen Multiplex PCR kit (Qiagen). The final concentrations of reagents in a total volume of 10 μl were as follows: 1X Qiagen Multiplex PCR Master Mix; 160 nM *IAP*‐F, *IAP*‐R, and *prefoldin‐*F; 320 nM *prefoldin*‐R and VICtail fluorescent tag; and 1 μl of template DNA. PCR was performed twice for each larva, once using gDNA and once using cDNA as the template. Thermocycling conditions consisting of 95°C for 15 min, 30 cycles of 94°C for 30 s, 56°C for 90 s, and 72°C for 60 s, and a final extension step at 60°C for 30 min.

Fragment analysis was performed on each of the PCR products using ABI 3130xL with GeneScan 500 LIZ size standard, scored using GeneMapper v4.0 (Life Technologies) and visually inspected to determine any miscalled alleles. Many cDNA samples were manually scored because differences in peak heights between alleles resulted in miscalled alleles. The electropherograms produced by gDNA and cDNA from each individual were compared. The ratios of the peak heights representing the neo‐X and neo‐Y alleles of *IAP* were compared between amplicons produced from gDNA and cDNA.

### 2.7 Relative expression of *IAP* in different genotypes using RT‐qPCR

The cDNA synthesized from Tête Juane Cache larvae was used as the template for RT‐qPCR using an ABI 7300 system. *IAP* was the target gene, and *actin*,* porphobilinogen deaminase* (*PBD*), and *RNA polymerase II* (*RPII*) were used as reference genes. These reference genes were previously identified by Fraser et al. (2017) as effective for normalizing gene expression in *D. ponderosae. *Each time‐point was used as a separate experiment to account for any temporal changes in expression. The 3 October 2008 time‐point experiment was conducted using 26 larvae and two reference genes, *actin* and *RPII*. Reactions contained final concentrations of 1X TaqMan Environmental Master mix, 0.4 μM of each primer, 0.1 μM of TaqMan hydrolysis probe, and 1 ng of cDNA in a final volume of 25 μl. The 10 October 2008 time‐point experiment was conducted using 28 larvae and all three reference genes. Reactions contained final concentrations of 1X TaqMan Fast Advanced Master Mix, 0.5 μM of each primer, 0.25 μM TaqMan hydrolysis probe, and 1 ng of cDNA template in a total volume of 20 μl (Table 2).

**Table 2:** The sequences for the primers and probes for the target gene (*IAP*) and the three reference genes (*Actin*,* PBD*,* and RPII*) used for RT‐qPCR of overwintering *D. ponderosae* larvae. For each probe, the reporter dye and the quencher used are reported

Gene	Primer/probe	Primer sequence (5′‐3′)
Target gene		
* Inhibitor of apoptosis*	*IAP‐*F	CCGAGAGATCGCCAAGGA
	*IAP*‐R	CGCACACTGGACAATATCACTTTC
	*IAP‐*probe	VICN‐TGCTGGCAAAGGCTGGATTCTATTACAAAA‐TAMRA
Reference genes		
* Actin*	*Actin‐*F	AAATTTTAACCGAACGTGGATATTC
	*Actin‐*R	CATCTCCTGTTCAAAGTCCAGAG
	*Actin‐*probe	JOEN‐TCACCACCACTGCCGAAAGGGAA‐BHQ‐1
* Porphobilinogen deaminase* (*PBD*)	*PBD‐*F	GGCTTCAATGTGTGTCCAGTG
	*PBD*‐R	CACCAAACCAACGAAAAGATGTTC
	*PBD‐*probe	JOEN‐CGCCAATCTTATCACCGTTGCCG‐BHQ‐1
* RNA polymerase II* (*RPII*)	*RPII‐*F	GACGTTGGAGCAGTTCAAAGAG
	*RPII‐*R	GGAAGAACACGAACATCTGGTC
	*RPII‐*probe	JOEN‐CGGCAAACCCAGCGAGAAGAGGC‐BHQ‐1

For each target and reference gene, a standard curve was produced using three technical replicates of serially diluted pooled cDNA and used to optimize the conditions to obtain high R^2^ values, efficiencies between 90 and 100%, and to determine concentrations of cDNA template within the linear dynamic range as recommended by Bustin et al. (2009). Therefore, the thermocycling conditions were different for some of the genes used; however, they all had 45 cycles of denaturation and annealing/extension (Table 3).

**Table 3:** The thermocycling conditions after optimization for the target gene *inhibitor of apoptosis* (*IAP*), and the three reference genes *actin*,* porphobilinogen deaminase* (*PBD*), and *RNA polymerase II* (*RPII*) all using 45 cycles of denaturation and anneal/extension

Date	October 3, 2008		October 10, 2008					
Step	All genes		*IAP* and *actin*		*PBD*		*RPII*	
	Temp (°C)	Time	Temp (°C)	Time (s)	Temp (°C)	Time (s)	Temp (°C)	Time (s)
UNG incubation	50	5 min	50	120	50	120	50	120
Polymerase activation	95	10 min	95	20	95	20	95	20
Denature	95	30 s	95	10	95	10	95	10
Anneal/extension	55	1 min	56	30	58	45	56	60

Individuals were divided according to genotype into four groups: females homozygous for 162, females heterozygous with a 162 allele, males with a 162 neo‐X allele, and males lacking a 162 neo‐X allele. The data obtained from RT‐qPCR were analyzed using REST2009 software (Qiagen; Pfaffl, Horgan, & Dempfle, 2002) to determine the relative expression of *IAP* between different genotypes and also between males and females.

## 3 RESULTS

### 3.1 Gene‐linked microsatellite databases

#### 3.1.1 Sex linkage of gene‐linked microsatellites

In the six Western Canadian populations genotyped, sex linkage was noted for three of the 16 gene‐linked loci when compared to a previously identified genetic marker for sex linkage (Davis et al., 2009). Two of these loci, MPBC6_675 (*IAP*) and MPBC6_7245 (*prefoldin subunit 5*) had distinct sets of neo‐X and neo‐Y alleles. The third locus, MPBC8_12800 (*antennae‐rich cytochrome P450*), only amplified from male samples and was therefore excluded from further analysis. The sex linkage of *IAP* was consistent in beetles from 52 North American populations genotyped.

#### 3.1.2 Signatures of selection

**Figure 2:**
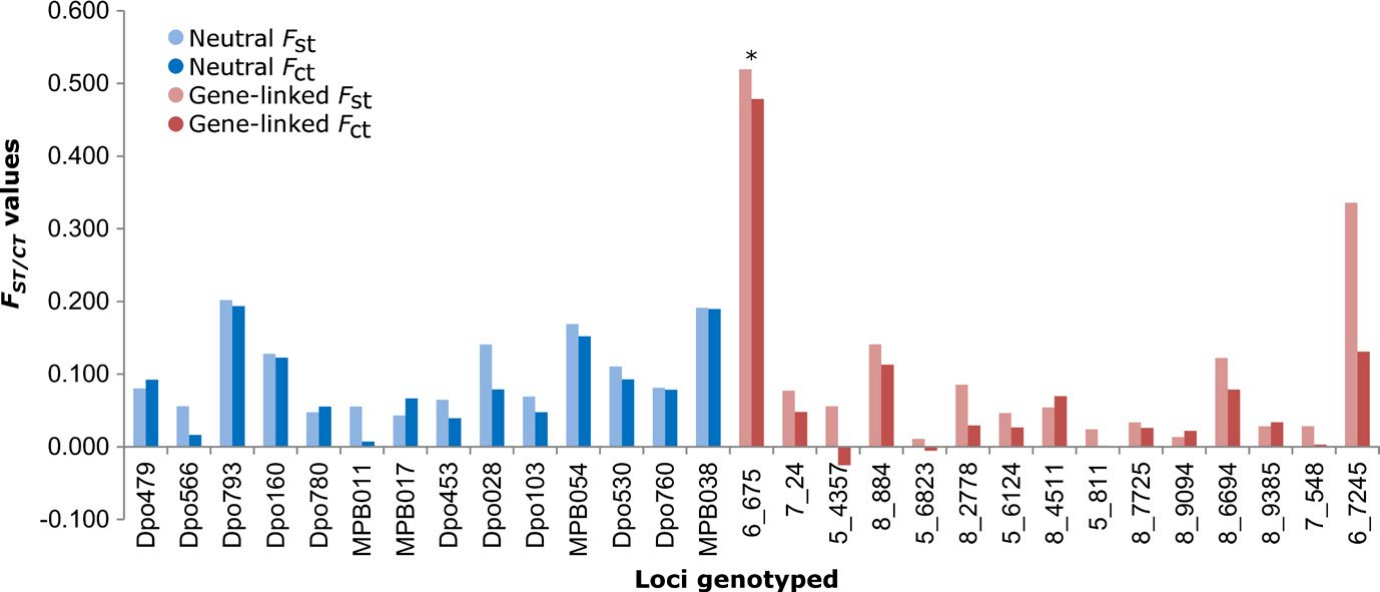
The *F*_st_ and *F*_ct_ values for neutral and gene‐linked microsatellites genotyped in mountain pine beetles collected in Western Canada. * indicates an outlier based on both *F*_st_ and *F*_ct_ values (*p* < 0.01)

The Western Canada microsatellite database with six sampling locations (Supporting Information Table S1) was divided into male and female datasets to eliminate any influence that the sex linkages of microsatellites could have on the identification of outliers. The female‐only dataset identified the trinucleotide repeat in the *IAP* coding sequence as the only significant outlier among 29 loci based on both the *F*_st_ values and *F*_ct_ values after accounting for population structure (Figure 2). *IAP* also showed a signature of diversifying selection in Western Canada after analysis using Bayescan (*α* = 1.553; *q*‐value = 0.000). In a second dataset containing neo‐X *IAP* and 14 neutral microsatellite loci in 52 North American population, *IAP* was again found to be a significant outlier based on *F*_st_ values (*F*_st_ = 0.437; *p* = 1e‐7), *F*_ct_ values (*F*_ct_ = 0.358, *p* = 1e‐7) and show a signature of diversifying selection using BayeScan (*α* = 0.110; *q*‐value = 0.048).

#### 3.1.3 Analysis of spatial distribution of *IAP* alleles

**Figure 3:**
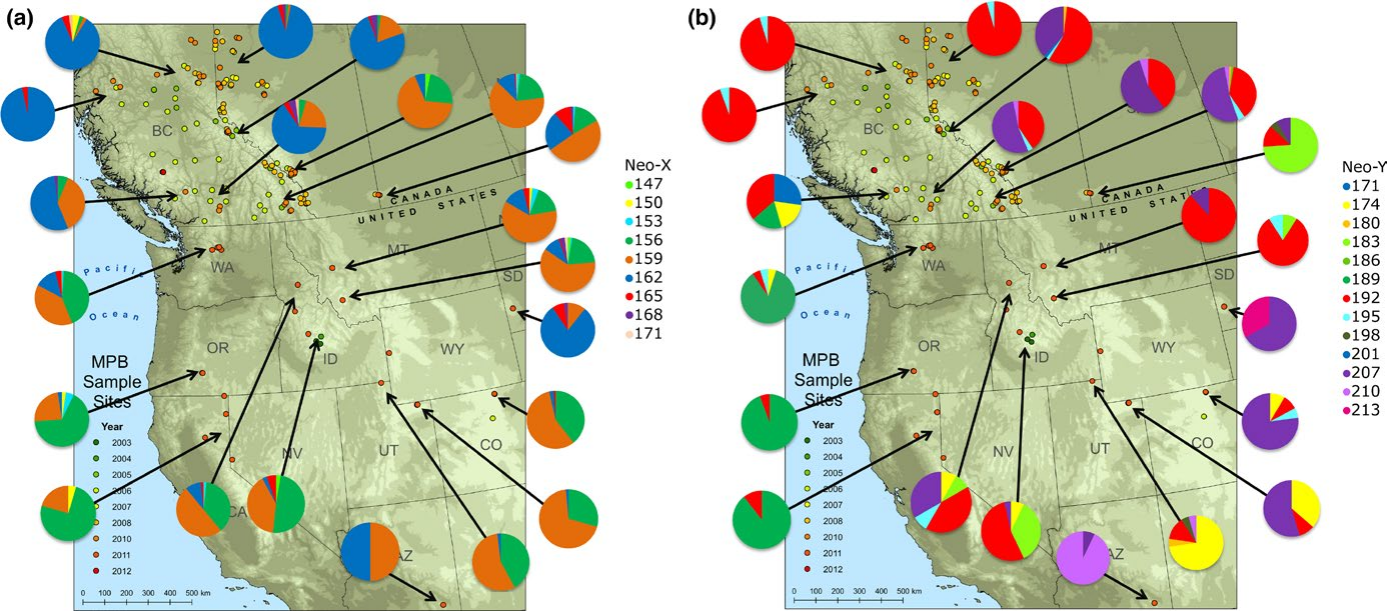
Spatial distribution of (a) neo‐X alleles and (b) neo‐Y alleles of *IAP* genotyped in mountain pine beetles collected from the indicated sampling locations across western North America

The frequencies of both neo‐X and neo‐Y *IAP* alleles in North American sampling locations were determined and trends were inspected. The 162‐bp allele, corresponding to a 14 serine repeat, was the most common neo‐X allele in the north and was near fixation in some northern populations. In the central and southern ranges, the 156‐bp and 159‐bp alleles are the most common neo‐X alleles and there is more of a mixture of genotypes; however, there are locations in South Dakota and Arizona where a 162‐bp allele is common (Figure 3). The neo‐Y specific alleles show more variation in the general clusters of allele frequencies than the neo‐X alleles. Some populations have a greater mix of neo‐Y alleles, and isolated populations such as Cypress Hills, Arizona, and South Dakota have otherwise uncommon neo‐Y alleles (Figure 3).

#### 3.1.4 Neo‐X and neo‐Y allele sequences

**Figure 4:**
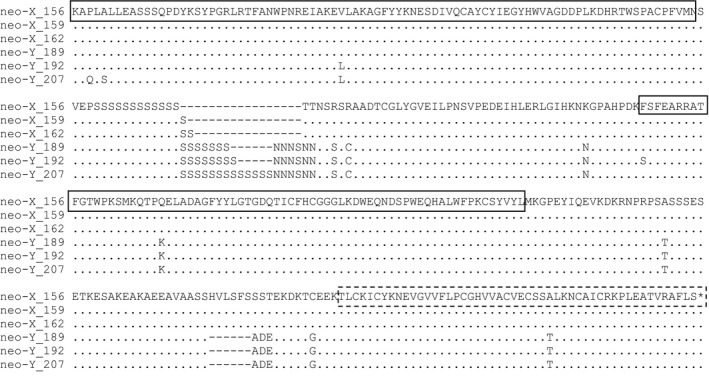
An alignment of the translated sequences of the *inhibitor of apoptosis* (*IAP*)156, 159, and 162‐bp neo‐X alleles; and 189‐, 192‐, and 207‐bp neo‐Y alleles. The amino acids differing from the 156‐bp allele are indicated, whereas the conserved amino acids are represented as dots and gaps as dashes. The conserved domains are indicated as follows: the two BIR domains are boxed with a solid line, whereas the RING domain is boxed with a dashed line

Among the commonly observed neo‐X alleles of *IAP* sequenced (156, 159, and 162), there was no amino acid variation outside of the microsatellite region (Figure 4). Variation was greater among the neo‐Y alleles and between the neo‐X and neo‐Y alleles. In particular, there were two indels in the alignment of the neo‐X and neo‐Y sequences. The first indel region is associated with the trinucleotide (serine) repeat and lies in a region between two predicted BIR domains. A second indel region lies between the predicted BIR2 and RING domains. The dN/dS ratio between the 162‐bp neo‐X and 192‐bp neo‐Y alleles was 0.515 and a codon‐based *Z*‐test failed to reject neutral evolution (*z* = −1.44, *p* = 0.152). Four of the nonsynonymous changes resulting in amino acid changes were in the BIR2 and RING predicted domains.

### 3.2 Expression of different *IAP* Alleles

#### 3.2.1 Genotyping of individuals

Prior to analysis of expression, individual beetles were genotyped from both gDNA and cDNA using the sex‐linked microsatellites in *IAP* and *prefoldin subunit 5* and were assigned into four groups based on their genotype. They were genotyped from both gDNA and cDNA to verify results because in some males from Utah, California, and Arizona the peak height of the neo‐Y allele of *IAP* was not discernible above background when genotyped from cDNA alone due to a lack of expression.

#### 3.2.2 Qualitative relative expression of *IAP* alleles

**Figure 5:**
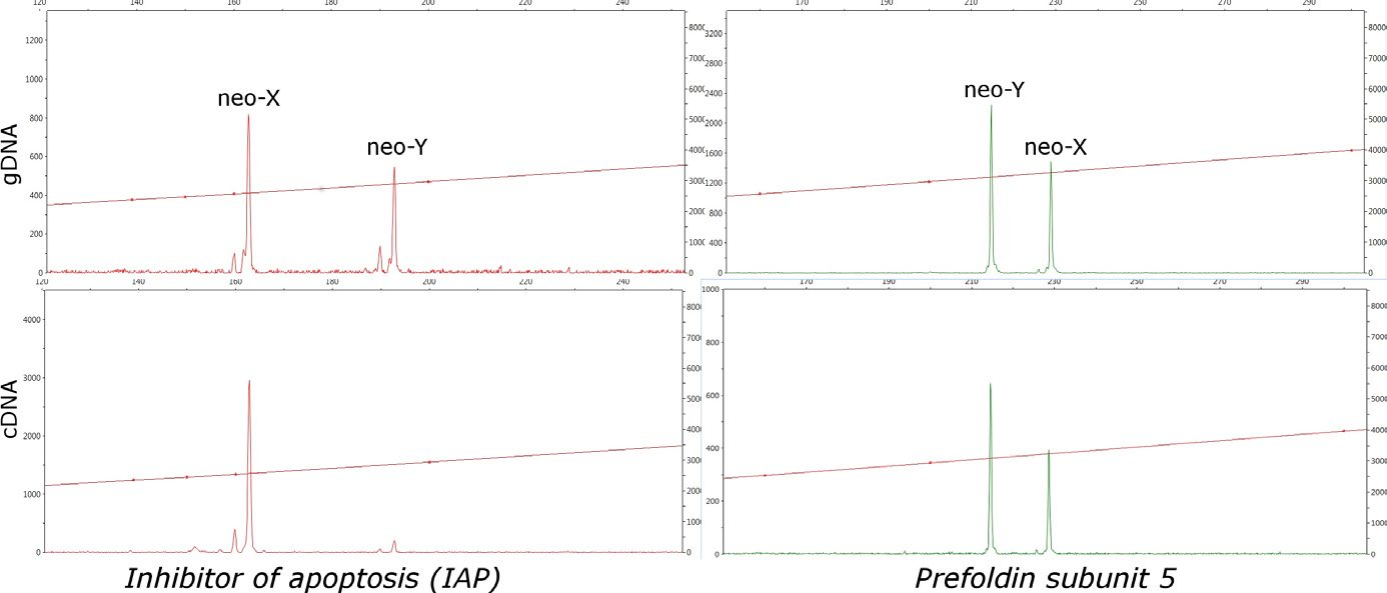
The peak heights of neo‐X and neo‐Y alleles of both *IAP* and *prefoldin subunit 5* amplified from gDNA compared to the same alleles amplified from cDNA from a single individual larva. The neo‐Y allele of *IAP* amplified from cDNA is greatly reduced compared to the allele amplified from gDNA, but no difference is observable for the neo‐Y allele of *prefoldin subunit 5*

The electropherograms from the amplification of *prefoldin subunit 5* produced peaks with equal neo‐X to neo‐Y peak height ratios from gDNA and cDNA. In contrast, the electropherograms from the amplification of *IAP* from cDNA produced peaks with higher neo‐X to neo‐Y peak height ratios than those from gDNA (Figure 5). Based on the electropherograms from 18 male samples from Tête Juane Cache, BC the mean ratio of neo‐X to neo‐Y allele peak heights from cDNA was an average of 9.55 times higher than the ratio from gDNA (*p* < 0.005). A similar trend was found in all male individuals from Utah, California, Arizona, and Cypress Hills. In each location, the peak produced by the neo‐Y allele was reduced in the cDNA compared to the gDNA. However, the ratio was not quantifiable since the peak corresponding to the neo‐Y allele was not visible above background in many of the electropherograms.

#### 3.2.3 Quantitative relative expression of *IAP* in larvae

**Figure 6:**
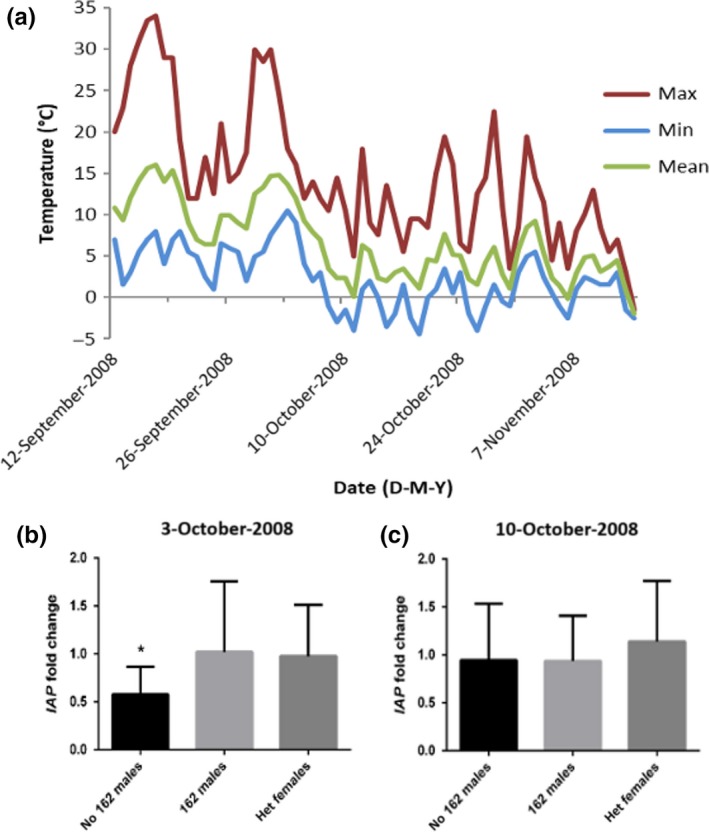
(a) The temperature data from September to November of the year larvae were sampled is shown. (b, c) The relative expression of *IAP* in larvae with different genotypes: males lacking the 162‐bp allele (no 162 males); males with the 162‐bp allele (162 males), and females heterozygous for the 162‐bp allele, compared to the expression of *IAP* in females homozygous for the 162‐bp allele. At the 3 October 2008 time‐point corresponding to a drop in temperature the male larvae lacking the 162‐bp allele had significantly less *IAP* expression than the females homozygous for the 162‐bp allele (b). By 10 October 2008, the differences in expression were no longer significant (c)

The optimized conditions for RT‐qPCR using cDNA from Tête Juane Cache resulted in standard curves having high R^2^ values and efficiencies over 90%. The relative expression of *IAP* in males lacking the 162‐bp neo‐X allele was reduced to 0.577‐fold of expression in females homozygous for the 162‐bp allele at the 3 October 2008 time‐point (*p* = 0.038). At the 10 October 2008 time‐point, there were no significant differences in *IAP* expression between any of the other genotypes compared to the females heterozygous for the 162‐bp allele (Figure 6). None of the female beetles sampled lacked the 162‐bp allele and so we were unable to make any comparisons with females lacking the 162‐bp allele altogether. The relative expression of *IAP* was also not significantly different between males and females at the 3 October 2008 time‐point (expression ratio = 0.883; *p* = 0.289) or the 10 October2008 time‐point (expression ratio = 0.958; *p* = 0.785). Finally, the overall *IAP* expression among all larvae was found to increase significantly 1.331‐fold (*p* = 0.013) from the 3 October to 10 October time‐point.

## 4 DISCUSSION

The inhibitor of apoptosis (*IAP*) gene was identified as a candidate for diversifying selection using a database of neutral and gene‐linked microsatellites and multiple outlier analyses. The neo‐X alleles of *IAP* were consistently identified as outliers based on *F*_st_ values, *F*_ct_ values (Figure 2), and Bayesian approaches. Upon inspection of the allele frequencies of the neo‐X *IAP* alleles across North America, the 162‐bp allele was noted to be the most common allele in the northernmost range as well as in some of the southernmost regions (Figure 3). Therefore, we hypothesized that the 162‐bp allele may be providing a local adaptation to beetles experiencing harsh conditions, including extreme cold in the north. Assuming that the southern 162‐bp allele is orthologous to the northern 162‐bp allele, this allele may be helping not only with cold tolerance, but more generally with adapting to stresses at the limits of *D. ponderosae*'s geographical range.

A wide range of neo‐Y alleles was genotyped across North America and the distribution of alleles did not closely follow the distribution of neo‐X alleles (Figure 3) suggesting an independent evolution of neo‐Y alleles. Recent findings suggest that the neo‐Y chromosome in *D. ponderosae* forms three distinct geographically isolated SNP haplogroups (Bracewell, Bentz, Sullivan, & Good, 2017; Dowle et al., 2017). Using this single gene, we find consistency with the populations from the western United States being similar; however, in Canada, eastern US, and central US, we find more differences among neo‐Y alleles within populations than would be predicted by geographic range of the SNP haplogroups (Figure 3b). This suggests that the inclusion of neo‐Y microsatellite markers with SNPs (Bracewell et al., 2017; Dowle et al., 2017) will provide a finer resolution of within haplogroup variation which can inform evolution of the neo‐Y chromosome in these areas.

The independent evolution of neo‐X and neo‐Y alleles in *D. ponderosae* is of particular interest because there has been a recent fusion between an ancestral autosomal and X chromosome as well as the loss of the ancestral Y chromosome (Keeling et al., 2013; Lanier & Wood, 1968). This is informed by comparison with other *Dendroctonus* species which have 12 autosomal chromosomes and two sex chromosomes (X and Y), whereas *D. ponderosae* and *D. jeffreyi* have 11 autosomal chromosomes and two sex chromosomes (neo‐X and neo‐Y) (Zúñiga et al., 2002). It is expected that over time sequence divergence will occur between the ancestral autosomal portions of the neo‐X and neo‐Y chromosomes explaining the distinct *IAP* alleles observed. However, it is still early in this divergence so we were able to amplify and study both the neo‐X and neo‐Y alleles with common primer sets.

Analysis of sequence variation between the neo‐X and neo‐Y *IAP* alleles failed to reject neutral evolution. This variation suggests that the neo‐Y *IAP* may no longer be evolving under functional constraints indicating that it may not contribute substantially to overall IAP production. Furthermore, when male samples were genotyped the electropherograms for *IAP* amplified from cDNA had a reduced peak height produced by the neo‐Y allele. The peak height ratio of the *IAP* neo‐X allele compared to the neo‐Y allele was consistently larger in the electropherograms produced from amplification of cDNA than those from gDNA across all populations, representing seven different neo‐Y alleles (Figure 5). As the ratio of neo‐X to neo‐Y alleles in genomic DNA is one to one, this suggests that there were more neo‐X allele transcripts of *IAP* than neo‐Y allele transcripts to acts as templates for cDNA synthesis due to reduced expression of the neo‐Y allele. Reduced expression may have led to the loss of functional constraints which resulted in sequence variation between the neo‐Y and neo‐X alleles being consistent with neutral evolution.

In other insects, such as *D. miranda* with neo‐X/neo‐Y sex chromosomes, the neo‐Y chromosome is degenerating (Bachtrog & Charlesworth, 2002). Based on the sequence variation between the *IAP* neo‐X and neo‐Y alleles presented in this study, there is likely degeneration in the *D. ponderosae* neo‐Y chromosome. This is supported by recent findings that there has been degeneration in the neo‐Y chromosome of *D. ponderosae*. Bracewell et al. (2017) report large deletions in the neo‐Y chromosome suggesting degeneration, whereas we find divergence usually associated with a pseudogene (equal number of synonymous and nonsynonymous changes) within a single gene between the neo‐X and neo‐Y chromosomes. These pieces of evidence point to neo‐Y degeneration which may provide a highly selective environment for the orthologous neo‐X alleles. Outside of the variable number of serine repeats, there was no sequence variation in the coding sequences of neo‐X alleles analyzed, suggesting that differences in protein activity of different neo‐X alleles are unlikely. Therefore, potential expression differences among neo‐X alleles were examined.

Overall *IAP* expression among all larvae was found to increase significantly from 3 October to 10 October consistent with an overall trend of increasing *IAP* expression in larvae preparing for overwintering which had a 3.13‐fold increase from 26 September to 7 November 2008 in the same collection (Robert et al., 2016). This increase in expression as the larvae prepare for overwintering suggests that *IAP* may play a role in cold tolerance. This potential role in cold tolerance is supported by the role of *IAP* as a caspase inhibitor as apoptosis involving caspases has been shown to be inducible by cold shock (Fransen, Dieker, Hildebrands, Berden, & van der Vlag, 2011). Furthermore, in *Drosophila melanogaster*, an inhibitor of apoptosis Bcl‐2 was shown to be upregulated at the protein level during rapid cold‐hardening to compensate for dysregulated apoptosis which is common during cold shock (Yi, Moore, & Lee, 2007) Therefore, elevated levels of *IAP* expression may help the larvae to survive overwintering.

The relative expression of *IAP* in overwintering larvae was compared for beetles with varying genotypes. Individuals lacking a 162‐bp neo‐X allele were of interest as this allele was most common in the north. Among the larvae sampled on 3 October males lacking the 162‐bp allele had reduced *IAP* expression compared to females homozygous for the 162‐bp allele. However, among the 10 October samples, the expression of *IAP* was not significantly different in any group of larvae compared to females homozygous for the 162‐bp allele (Figure 6). The reduced expression of *IAP* in males lacking the 162 allele at the 3 October time‐point, but equal expression at the 10 October time‐point might reflect a more efficient or rapid upregulation of the 162‐bp *IAP* allele. Earlier expression of this *IAP* allele may give those larvae increased survival during early extreme cold events which would be more likely to occur earlier in the northeastern part of the beetle's range thereby providing a selective advantage. Unfortunately, due to the high frequency of the 162‐bp allele in this population, it was not possible to examine all allele combinations and compare with females completely lacking this allele.

The lack of expression differences between males and females suggests that dosage compensation may be occurring. As discussed, it appears that the neo‐Y alleles have reduced expression based on the electropherograms from cDNA. Therefore, dosage compensation would be necessary to allow equal expression of *IAP* in males and females. Although some form of dosage compensation is known to occur in many species with both neo‐X/neo‐Y and X/Y sex chromosomes, it has not been reported in bark beetles. *D. miranda* has a neo‐X/neo‐Y sex chromosome system which has dosage compensation partly developed for neo‐X alleles in males (Bachtrog & Charlesworth, 2002). In particular, there is increased expression of genes on the neo‐X chromosome which have degenerated orthologous genes on the neo‐Y chromosome (Marín, Siegal, & Baker, 2000). In *D. ponderosae,* the sequence variation and reduced expression suggests that the neo‐Y *IAP* allele may be degenerating. Furthermore, another sex‐linked gene, *prefoldin subunit 5*, did not show variation in the peak heights of neo‐X and neo‐Y alleles. This is also consistent with the neo‐X/neo‐Y dosage compensation in *D. miranda* which is not chromosome wide (Bone & Kuroda, 1996).

Although there is little known about dosage compensation in Coleoptera, Prince, Kirkland, and Demuth (2010) found that the majority of X‐linked genes in the red flour beetle, *Tribolium castaneum,* have increased expression in females. As the X‐linked genes in male flour beetles were expressed at approximately the same levels as autosomal genes, the increased expression of X‐linked genes in females is believed to be an evolutionary side effect of an imperfect dosage compensation mechanism. This hyperexpression of X‐linked genes shows that differential expression of sex chromosomes occurs in Coleoptera. However, the equal expression of *IAP* in male and female *D. ponderosae* suggests that, in contrast to *T. castaneum,* it is unlikely that neo‐X linked genes are globally hyperexpressed in females. It is possible that *D. ponderosae* is more similar to *Xenos vesparum* in which the chromosomal segment corresponding to the ancestral X shows dosage compensation but dosage compensation in the recently added segment of the X chromosome is incomplete (Mahajan & Bachtrog, 2015). However, the expression of more neo‐X linked genes particularly those in the ancestral X segment would need to be determined to make a definite conclusion on the extent and mode of dosage compensation in *D. ponderosae*.

Despite recent studies on sex chromosome evolution in *D. ponderosae* (Bracewell et al., 2017; Dowle et al., 2017), there is still little known about dosage compensation and much to be explored on a finer scale regarding sex chromosome evolution in bark beetles, so studying variation in both the sequence and expression of neo‐X and neo‐Y linked genes can help determine how the sex chromosomes are evolving and predict how they will continue to evolve. The sequence variation between the *IAP* neo‐X and neo‐Y alleles suggests that the neo‐Y chromosome is degenerating in *D. ponderosae*. Such degeneration fosters a highly selective environment for neo‐X alleles as they must compensate for an orthologous neo‐Y allele with a reduced or lost function. In the case of *IAP*, the 162‐bp allele appears to have faster upregulation compared to the other neo‐X alleles, which could provide a selective advantage allowing mountain pine beetle larvae in the north to survive early cold snaps.

The sequence variation and expression differences in mountain pine beetle *IAP* suggest that the neo‐Y allele is not contributing substantially to the overall production of the protein. However, relative expression of *IAP* in larvae preparing for overwintering shows that males do not have lower expression levels except for males lacking the northern 162‐bp allele at the earlier time‐point. This suggests that some mechanism of dosage compensation may be controlling the expression of this sex‐linked gene in mountain pine beetle. Further, the single functional copy of *IAP* in males would provide a highly selective environment for any alleles with earlier upregulation. Future research should investigate more sex‐linked genes that may be providing local adaptation and expression differences between males and females. This line of research could refine when expression differences occur in genes important for overwintering, provide insights into the mechanism of dosage compensation, and potentially explain the female skewed sex ratio in the adult population. This could help identify loci under strong selection that may explain how populations are expanding successfully into novel habitats northeast of the historic range.

## CONFLICT OF INTEREST

None declared.

## AUTHOR CONTRIBUTIONS

The experiments were designed and performed by LCH, CKB, GS, GKK, and BWM. Analysis of the data for this manuscript, preparation of the figures, and the manuscript were conducted by LCH and BWM. CKB, GS, and GKK critically reviewed the manuscript.
