# Supplementary material for: Selection of the sex‐linked inhibitor of apoptosis in mountain pine beetle (Dendroctonus ponderosae) driven by enhanced expression during early overwintering
Source: Ecol Evol. 2018 May 24;8(12):6253–64. doi: 10.1002/ece3.4164 (PMC6024124; doi:10.1002/ece3.4164)
Supplement: Supplementary file 1 [file ECE3-8-6253-s001.docx]

| Locus | Number of alleles | Observed Heterozygosity | Expected Heterozygosity | Fixation Index (Fst) |
| --- | --- | --- | --- | --- |
| Dpo479_1 | 3.83 | 0.722 | 0.672 | 0.068 |
| Dpo566_1 | 2.67 | 0.248 | 0.234 | 0.071 |
| Dpo793_1 | 4.00 | 0.523 | 0.491 | 0.138 |
| Dpo160_1 | 6.17 | 0.720 | 0.685 | 0.094 |
| Dpo780_1 | 3.67 | 0.529 | 0.571 | 0.049 |
| MPB011_1 | 3.67 | 0.618 | 0.548 | 0.073 |
| MPB017_1 | 3.00 | 0.392 | 0.402 | 0.044 |
| Dpo453_1 | 5.00 | 0.695 | 0.686 | 0.072 |
| Dpo028_1 | 4.00 | 0.453 | 0.418 | 0.119 |
| Dpo103_1 | 7.50 | 0.828 | 0.782 | 0.072 |
| MPB054_1 | 2.17 | 0.200 | 0.195 | 0.130 |
| Dpo530_1 | 4.00 | 0.592 | 0.623 | 0.093 |
| Dpo760_1 | 5.33 | 0.567 | 0.590 | 0.070 |
| MPB038_1 | 3.17 | 0.251 | 0.267 | 0.136 |
| MPBC8_2778 | 1.83 | 0.087 | 0.128 | 0.089 |
| MPBC5_6124 | 3.67 | 0.573 | 0.645 | 0.061 |
| MPBC8_4511 | 7.83 | 0.735 | 0.766 | 0.051 |
| MPBC6_7245 | 2.17 | 0.087 | 0.237 | 0.291 |
| MPBC5_811 | 4.17 | 0.705 | 0.677 | 0.051 |
| MPBC8_7725 | 2.67 | 0.220 | 0.200 | 0.049 |
| MPBC8_9094 | 1.83 | 0.123 | 0.128 | 0.032 |
| MPBC8_6649 | 3.33 | 0.512 | 0.570 | 0.109 |
| MPBC8_9385 | 4.33 | 0.485 | 0.451 | 0.044 |
| MPBC7_548 | 3.17 | 0.575 | 0.581 | 0.052 |
| MPBC7_24 | 4.00 | 0.485 | 0.544 | 0.076 |
| MPBC5_4357 | 4.00 | 0.398 | 0.587 | 0.082 |
| MPBC8_884 | 3.17 | 0.413 | 0.435 | 0.116 |
| MPBC5_6823 | 1.67 | 0.058 | 0.072 | 0.040 |
| MPBC6_675 | 3.50 | 0.339 | 0.335 | 0.403 |

Supplemental Table 1. Summary of statistics for the female beetles for each locus averaged over the six Western Canadian populations. Heterozygosities and fixation indices were calculated using GenAlEx v6.5.
