# Supplementary material for: Selection of the sex‐linked inhibitor of apoptosis in mountain pine beetle (Dendroctonus ponderosae) driven by enhanced expression during early overwintering
Source: Ecol Evol. 2018 May 24;8(12):6253–64. doi: 10.1002/ece3.4164 (PMC6024124; doi:10.1002/ece3.4164)
Supplement: Supplementary file 2 [file ECE3-8-6253-s002.docx]

Supplemental Table 2. The GenBank accession numbers for the gene-linked microsatellites used in this study are listed as well as the scaffolds each gene is assembled on. Each of these scaffolds is unplaced and the chromosomes are unknown.

| Microsatellite | Accession number | Scaffold |
| --- | --- | --- |
| MPBC8_7725 | GT436798 | NW_017851171.1 (531562 - 537571) |
| MPBC5_6124 | GT403944 | NW_017851689.1 (187950-189458) |
| MPBC5_811 | GT357891; | NW_017851724.1 (60522-63937) |
| MPBC6_675 | GT401041; GT408450 | NW_017851754.1 (1976-55801); NW_017846291.1 (715-2328) |
| MPBC6_7245 | GT339861 | NW_017848488.1 (396-2219) |
| MPBC7_548 | GT320845 | NW_017852003.1 (952602-1003666) |
| MPBC8_2778 | GT344705 | NW_017852017.1 (1379610-1394281) |
| MPBC8_4511 | GT415941 | NW_017852336.1 (576423-577619) |
| MPBC8_6649 | GT419741 | NW_017852298.1 (41309-58204) |
| MPBC8_9094 | GT433817 | NW_017852126.1 (1530033-1531537) |
| MPBC8_9385 | GT451465 | Not mapped |
| MPBC5_6823 | GT404280 | Seq_985438 (c53177-48196) |
| MPBC8_884 | GT421807 | NW_017851823.1 (296456-300234) |
| MPBC8_12800 | GT490735 | NW_017852171.1 (28913-32226) |
|  |  |  |
| MPBC5_4357 | GT429515 | NW_017851564.1 (38801-39590) |
| MPBC7_24 | GT317345 | NW_017852120.1 (51505-69946) |
